# Fungi Associated With Freshwater Zooplankton Are Taxon‐Specific, Temporally Dynamic and Reflect Allochthonous Inputs

**DOI:** 10.1111/mec.70436

**Published:** 2026-07-24

**Authors:** Johannes Schweichhart, Caio César Pires de Paula, Veronika Kreidlová, Jaroslav Vrba, Michal Šorf, Dagmara Sirová

**Affiliations:** ^1^ Institute of Hydrobiology, Biology Centre CAS České Budějovice Czech Republic; ^2^ Faculty of Science University of South Bohemia České Budějovice Czech Republic; ^3^ Faculty of AgriSciences Mendel University in Brno Brno Czech Republic

**Keywords:** Cladocera, Copepoda, microbiome, precipitation, Rotifera, saprotroph, spore size, terrestrial, yeast growth form

## Abstract

The associations between microbes and planktonic invertebrates in freshwater ecosystems are considered key ecological interactions. However, our understanding of the structure, taxonomic specificity, and environmental drivers of zooplankton microbiomes remains poor. Here, we present the results of a field study using ITS1‐amplicon sequencing that assesses the structure of fungal assemblages associated with three zooplankton groups (Cladocera, Copepoda and Rotifera) inhabiting the same water column during a single growing season. Compared with fungal communities in the surrounding water, zooplankton‐associated assemblages showed higher genus richness, particularly in Copepoda and Rotifera, but lower diversity and evenness. We identified 190 fungal genera spanning eight phyla, dominated by fast‐growing yeasts that thrive in nutrient‐rich environments and are commonly associated with soil, leaf litter and decaying wood. The taxonomic identity of the zooplankton emerged as the main factor shaping fungal assemblages, followed by spore size and precipitation. Fungal sequences in zooplankton samples were largely derived from taxa typically linked to soils and plants, and the community composition shifted after precipitation events, consistent with terrestrial inputs to the lake. No fungal taxa were found exclusively in zooplankton guts, suggesting that these associations are transient and trophic rather than colonising. Taken together, these patterns indicate that zooplankton feed on suspended fungal cells or spores, many of which originate from surrounding terrestrial habitats. This suggests an overlooked pathway connecting land and freshwater ecosystems, highlighting terrestrial fungi as an under‐recognised component of freshwater trophic linkages.

## 1 Introduction

Microbial associations with freshwater planktonic invertebrates—most often studied in small crustaceans such as water fleas—are considered key ecological interactions (Tang et al. 2010). However, our understanding of the structure, zooplankton species specificity, and environmental drivers of microbiomes, as well as their links to biogeochemical cycling in aquatic ecosystems, remains limited. There are two main types of direct associations between zooplankton and microbes: host–microbiome interactions (Akbar et al. 2022; Callens et al. 2018), including host–pathogen interactions (Duffy et al. 2010), and predator–prey interactions (for a review, see Kiørboe 2011).

In terms of the non‐pathogen host–microbiome relationships, the main niches available for microbial colonisation of zooplankton are the external bodily surfaces (Eckert and Pernthaler 2014) and the gut epithelia (Xu et al. 2024; Shoemaker and Moisander 2017). The composition and ecology of bacterial consortia associated with zooplankton surfaces (epibionts; Carman and Dobbs 1997) and colonising their digestive tracts (Shoemaker and Moisander 2017) have been relatively well studied. However, while bacterial associations with zooplankton have been studied in depth—ranging from transient surface colonisers to more stable taxon‐specific microbiomes—the role of fungi in such interactions remains largely unexplored (Bickel et al. 2012).

Research on freshwater zooplankton reveals that their bodily surfaces are hotspots for bacterial colonisation within the planktonic environment. Such bacterial assemblages are widespread across zooplankton taxa (Samad et al. 2020; Eckert et al. 2021), distinct from the surrounding water column (Samad et al. 2020), highly metabolically active (Eckert and Pernthaler 2014), and dynamic in both space and time (Eckert et al. 2021). The high temporal and environmental variability and high species turnover of these assemblages have been shown to result in a low proportion of ‘core’ microbial taxa (Eckert et al. 2021, 2022). Although bacteria and fungi differ substantially in their ecophysiology, they tend to occupy the same environments, share resources (Kramer et al. 2016), and form important cross‐kingdom networks within animal‐associated microbiomes (Pawlowska 2024). A few studies from marine settings indicate that zooplankton are not an exception, and fungal sequences have been detected in microbial communities associated with a range of microscopic marine invertebrates (Holt et al. 2022), including Cladocera (water fleas; Serandour et al. 2023).

With the exception of animal pathogens and zoosporic parasites of phytoplankton (Van den Wyngaert et al. 2022), fungi have traditionally been viewed as playing a limited role in the ecology of lakes and ponds. This view is rapidly changing due to advances in predominantly molecular approaches in aquatic microbial ecology (Grossart et al. 2022). Several studies have highlighted the importance and hidden diversity of aquatic fungi, implicating them in organic matter cycling and food‐web dynamics (for reviews, see Krauss et al. 2011; Grossart et al. 2019). Zoospores produced by parasitic fungi from the Chytridiomycota and Rozellomycota phyla are considered to be a good food source for zooplankton, and feeding on these zoospores (‘mycoloop’) has been proposed to be an important food web component within the planktonic environment (Kagami et al. 2014). In addition, fungal propagules from terrestrial systems enter freshwaters via atmospheric deposition and runoff (Abrego et al. 2024; Kołaczek et al. 2013) and may represent an additional nutrient source for zooplankton. Although zooplankton feeding on Ascomycota and Basidiomycota fungi and their spores has rarely been studied outside laboratory settings, several invertebrate groups are known to consume fungal hyphae or propagules in terrestrial ecosystems (Santamaria et al. 2023) and streams (Suberkropp 1992). Furthermore, culture‐based studies have demonstrated that zooplankton can survive and reproduce on yeast diets (e.g., Kang et al. 2006; Gómez et al. 2012).

Based on the results of the studies mentioned above, we formulated the following research questions and hypotheses:

1. Do freshwater zooplankton associate with fungal assemblages that differ from those in the surrounding water column? Hypothesis 1: Each zooplankton group associates with distinct fungal assemblages primarily by host taxonomic identity
2. Which environmental and biological factors structure zooplankton‐associated fungal communities? Hypothesis 2: Fungal assemblages associated with zooplankton are predominantly composed of terrestrial or semi‐aquatic taxa introduced via runoff or atmospheric deposition, leading to variation in community composition with environmental conditions, particularly recent precipitation.
3. Do fungi associated with zooplankton represent colonising microbiota or potential prey? Hypothesis 3a: Gut and body communities (for taxa where dissection is possible) do not differ strongly, consistent with ingestion or transient passage of fungal cells or spores, rather than persistent gut colonisation. Hypothesis 3b: As particle size is a key parameter underlying prey selection among zooplankton (for an overview, see Hansen et al. 1994), fungal propagule and cell size play a significant role in structuring zooplankton‐associated fungal assemblages.

We used a hypereutrophic shallow lake in southern Bohemia (Czechia) to test the above‐mentioned hypotheses and investigated the structure of fungal microbiomes associated with different zooplankton taxa (Cladocera, Copepoda and Rotifera) co‐occurring in the same water column over a single growing season using ITS1‐amplicon sequencing. Our focus here is on non‐pathogenic fungi, and only animals without visible symptoms of fungal infection were selected for analysis. Environmental factors were quantified using physical, chemical and biotic variables that form the standard limnological framework for interpreting planktonic and microbial community structure (Wetzel 2001; Lampert and Sommer 2007).

## 2 Material and Methods

### 2.1 Study Location and Sampling Design

**FIGURE 1:**
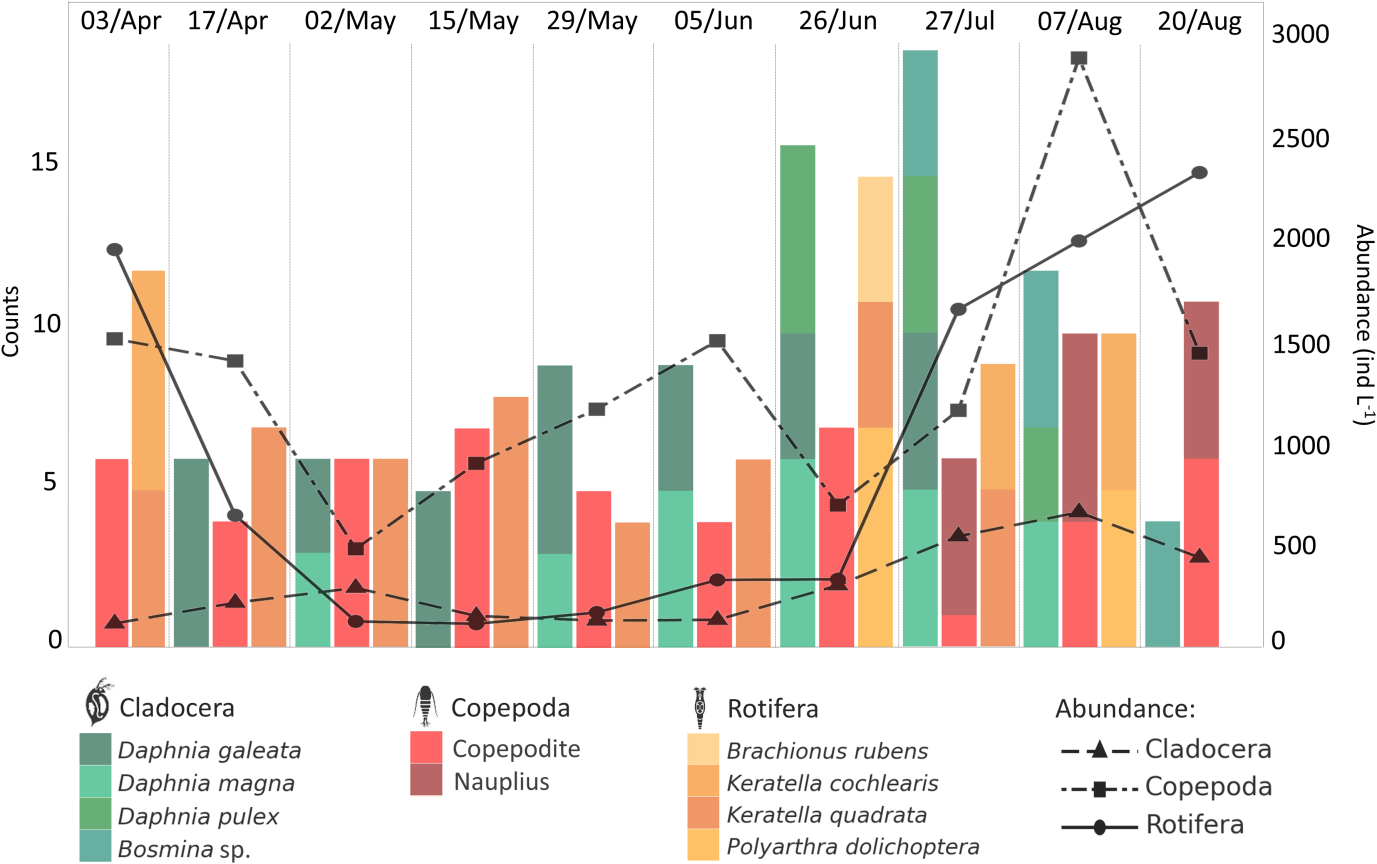
Number of individuals collected and successfully sequenced for each of the zooplankton species at each sampling point and abundance of the analysed zooplankton groups (individuals L^−1^) in the water column over the sampled period.

A shallow, man‐made hypereutrophic lake in South Bohemia, Czechia—the Kvítkovický fishpond (48.9633967° N, 14.3372900° E; WGS 84)—was sampled every 2 weeks from April to August 2019 (see Figure 1 for more details about sampling dates). The lake has an area of 24 ha, an average depth of 3 m and is representative of the thousands of fishponds established throughout central Europe during the Middle Ages, which are still managed by the fisheries authorities (Pechar et al. 2002; Vrba et al. 2024). The fishpond has functioned as a shallow lake ecosystem and an integral part of the landscape for several centuries and is part of a long‐term limnological research and monitoring program of shallow lakes, providing extensive accompanying environmental data (see Vrba et al. 2024, for details).

By integrating seven sampling points along a linear transect of the open water, we obtained a mixed water sample of the surface layer (50 L) collected using a Van Dorn sampler (1 m in length, with a 6.4 L volume, between 7 and 10 a.m.). All subsamples for plankton and chemical analyses were dispensed from the same mixed water sample. A subsample (ca. 3–5 L) was filtered through a 200‐μm mesh into a sterile plastic bottle to exclude zooplankton and large particles, and then processed for chemical analyses and DNA extraction in the laboratory within 2 h of collection. Zooplankton was concentrated by filtering a total volume of 30 L of the remaining mixed sample through sterile 200‐μm (larger crustaceans) and 40‐μm plankton nets (nauplii and rotifers). No specific sampling permits were required; permission to access the water body periodically was obtained from the relevant management authorities by the University of South Bohemia before sampling.

### 2.2 Environmental and Biotic Parameter Measurements

The following environmental parameters were measured as proxies for the physical, chemical and biotic conditions characterising the pelagic environment at each sampling point, according to standard protocols detailed in Vrba et al. (2024): temperature (°C), dissolved oxygen (mg L^−1^), Secchi depth (cm), conductivity (μS cm^−1^), chlorophyll *a* concentrations (μg L^−1^), total carbon (C), nitrogen (N) and phosphorus (P) concentrations (mg L^−1^), and seston molar ratios (C:P, C:N and N:P). Two‐day precipitation means were calculated from daily precipitation values programmatically extracted from the ERA5‐land reanalysis dataset using a Python script for the study period (Muñoz‐Sabater et al. 2021). In addition to the above parameters, the total numbers of bacteria and ciliates, phytoplankton biomass, and the proportions of different phytoplankton groups were recorded (see Table S1 for the environmental and biotic data used in this study).

### 2.3 Zooplankton Processing

Cladocera, Copepoda and Rotifera were immediately sorted in the lab under the dissecting microscope. Individuals were separated and identified to the lowest possible taxonomic level, then gently washed to remove larger debris through successive transfers along a series of sterile water washes. Individuals exhibiting signs of fungal infection or with hyphal growth on the surface of the body were discarded. Following the washing procedure, large Cladocera species (*Daphnia magna, Daphnia galeata* and *Daphnia pulex* ) were dissected, and the body and gut were separated into separate samples. All of the washed zooplankton samples were immediately stored at −20°C for further processing.

The total numbers of Cladocera, Copepoda and Rotifera per litre of the water column were quantified using the Sedgwick‐Rafter enumeration chamber. Only the nauplius and copepodite developmental stages of copepods were present and collected within the sampling period. Please see Figure 1 for the summary of zooplankton taxa and development stage density, as well as the total numbers of sequenced individuals belonging to the three sampled zooplankton groups present during each sampling point.

### 2.4 DNA Extraction and Sequencing

The DNA from single zooplankton individuals, or from body and gut samples in the case of *Daphnia* spp., was extracted using the Quick‐DNA Faecal/Soil Microbe Miniprep kit (Zymo Research, Irvine, CA, USA) according to the manufacturer's instructions.

Biomass for fungal assemblage analysis in the water column was collected from 0.3 to 0.8 L of sampled water (depending on turbidity), filtered onto polyethersulfone filters (0.22‐μm pore size, 47‐mm diameter; GPWP04700; Millipore‐Merck KGaA, Darmstadt, Germany) under aseptic conditions. The filters were stored at −80°C until extraction. The DNA was extracted by standard chloroform‐isoamyl alcohol extraction suitable for cells collected on membrane filters: TE‐saturated phenol at pH 8.0 (0.5 mL), 0.2 M sodium phosphate at pH 8.0 (0.5 mL) and 20% SDS (50 μL) were added to a lysing microbial matrix containing the collection filter. The tube was mechanically agitated, and DNA was extracted using chloroform‐isoamyl alcohol (24:1). The DNA was then precipitated with ethanol according to a standard protocol, resuspended in 50 μL of TE buffer, and stored at −20°C until further analysis.

DNA quantity and quality of fungal biomass from water‐column samples were assessed using a Qubit 3.0 fluorometer (Life Technologies) and a NanoDrop 2000 spectrophotometer (Thermo Fisher Scientific, Wilmington, DE, USA), with DNA concentrations ranging from 38 to 234 ng μL^−1^. In contrast, DNA concentrations in individual zooplankton samples were below the detection limits of these instruments, reflecting the hosts' microscopic size and the individual‐based extraction strategy (single gut/body/individual extractions). Despite DNA concentrations being below fluorometric detection limits, successful PCR amplification was obtained for all zooplankton samples, as confirmed by agarose gel electrophoresis, and yielded sufficient numbers of fungal sequence reads for downstream analyses (see below).

The internal transcribed spacer region 1 (ITS1) of the ribosomal operon was PCR‐amplified using ITS1F (5′‐CTTGGTCATTTAGAGGAAGTAA) and ITS2 (5′‐GCTGCGTTCTTCATCGATGC‐3′, White et al. 1990). The extracted DNA from water samples was amplified using the PPP Master Mix Polymerase 2× concentrated (TOP‐BIO, Czechia) in a final volume of 20 μL per sample (3 min at 94°C/30 cycles: 15 s at 94°C, 15 s at 60°C, 45 s at 72°C/10 min at 72°C), while for the zooplankton samples we used the FastStart Essential DNA Green Master (Roche, Czech Republic) following the same amplification conditions. Amplicons were sent to the Genomics and Microbiome Core Facility, Rush University Medical Center (Chicago, USA) for sequencing using Illumina MiSeq V2 (2 × 250) platforms (Illumina Inc., United States). Negative extraction and PCR controls were included at the relevant steps and were sequenced alongside the samples. The raw sequences were deposited in the European Nucleotide Archive (ENA) under the study accession number PRJEB94073.

### 2.5 Acquisition of Spore and Cell Sizes

Dimensions of fungal mitospores and meiospores at species level were taken from the github repository (Aguilar‐Trigueros et al. 2023). For yeast cell sizes, the means of at least three measurements were taken from the literature (Table S2).

### 2.6 Bioinformatic Processing and Data Analysis

ITS1 raw reads were quality‐checked using MultiQC (v. 1.27; Ewels et al. 2016) and processed using the PIPITS pipeline (v3.1; Gweon et al. 2015) with default settings and a clustering threshold of 97% for OTUs. SeqKit2 (v. 2.6.1; Shen et al. 2024) in combination with custom BASH and Python scripts was used to monitor and align the workflow. OTUs were taxonomically classified using BLASTn (‐evalue = 0.001; ‐word_size = 7; ‐reward = 1; ‐penalty = −1; ‐gapopen = 1; ‐gapextend = 2) and the UNITE database (v. 10.0; Abarenkov et al. 2024). Hits with *e*‐values higher than 1e^−20^ and coverage lower than 80% were removed, and taxonomic assignment was truncated based on the pident% scores of the blast‐hits (thresholds of 90%, 85%, 80%, 75%, 70% identity for genus‐, family‐, order‐, class‐, phylum‐level assignment, respectively). Contamination of commercial PCR reagents, including high‐sensitivity and hotstart polymerase kits, with fungal and other environmen‐tal DNA is a welldocumented phenomenon. This background DNA is amplified in ‐lowtemplate reactions, producing detectable reads even in negative controls. Because our single zooplankton samples contained very low DNA quantities, contamination is an important concern. To mitigate this, we applied stringent filtering and removed all OTUs detected in negative controls, which accounted for 219 of 12,794 OTUs (1.7%), ensuring that the remaining data reflect the true biological signal rather than reagent contamination. Related considerations led to our choice of primarily using presence‐absence and prevalence‐based downstream analyses, which are more robust towards these issues as detailed below. Downstream analysis mostly relied on phyloseq (v. 1.50; McMurdie and Holmes 2013), mia (v. 1.14; Borman et al. 2024), microviz (v. 0.12; Barnett et al. 2021), vegan (v. 2.6.8; Oksanen et al. 2024) R packages, custom Python and R scripts. Trait associations of fungal genera according to growth form (e.g., yeasts), primary lifestyle (e.g., nectar/tap saprotrophs) and aquatic potential (e.g., semi‐aquatic, non‐aquatic genera) were adopted from the FungalTraits database using the same terminology (v. 0.0.3; Põlme et al. 2020).

### 2.7 Statistical Analyses

To address the three hypotheses formulated in the Introduction, we used a complementary set of multivariate, univariate and differential‐abundance methods. Throughout, we used the conventional significance threshold of *α* = 0.05 for raw *p*‐values and *q* < 0.05 for false‐discovery‐rate‐adjusted *p*‐values, and unless stated otherwise, the default settings of the respective R/Python packages. Throughout the manuscript, the term *sample group* refers to the four‐level categorisation Cladocera/Copepoda/Rotifera/water column, and the term *zooplankton species* refers to the species‐level categorisation of zooplankton hosts (water column excluded).

#### 2.7.1 Multivariate Ordination of Community Composition (dbRDA)—Addresses Hypotheses 1 and 2

To visualise multivariate differences in fungal assemblage composition among zooplankton groups and the water column (Hypothesis 1) and to display the contribution of significant environmental covariates such as precipitation (Hypothesis 2), we used Distance‐based Redundancy Analysis (dbRDA; vegan::dbrda).

#### 2.7.2 Hypothesis Testing of Community‐Level Drivers (PERMANOVA)—Addresses Hypotheses 1, 2 and 3b

To formally test whether host taxonomic identity (Hypothesis 1), environmental covariates including two‐day precipitation (Hypothesis 2) and fungal spore size (Hypothesis 3b: size‐based prey selection) significantly structure the assemblages, we used PERMANOVA. PERMANOVA was chosen because it accommodates non‐normal multivariate community data and simultaneously partitions variance across multiple predictors. Models were fitted at two resolutions—sample group level (Cladocera, Copepoda, Rotifera, water column) and zooplankton species level—to assess the appropriate level of resolution at which fungal assemblages are structured. For both dbRDA and PERMANOVA, genus‐level OTU tables were transformed to presence/absence and analysed with Kulczynski distance to control for variation in DNA yield and low fungal biomass on individual specimens. Under this design, community‐level inferences depend on which fungal genera are detected and how often, not on absolute or relative read counts, which is more robust considering contamination risks and the low‐biomass single‐specimen libraries analysed here. Z‐score‐standardised library size (readsum) was included as a control variable to detect sequencing‐depth‐related artefacts. The full set of explanatory variables screened in PERMANOVA models included: host taxonomic identity, mitospore length, meiospore length, 2‐day precipitation, temperature, dissolved oxygen, Secchi depth, conductivity, chlorophyll a, total C, total N, total P, seston stoichiometry (C:P, C:N, N:P), bacterial and ciliate densities and sampling month/season. Continuous variables were *z*‐score‐standardised prior to fitting, and samples with missing values in any of the model variables were excluded using complete cases so that the sample group and zooplankton species models were fitted on identical sample sets. To assess robustness of the sample group PERMANOVA result to imbalanced sample sizes and dispersion heterogeneity, we additionally tested homogeneity of multivariate dispersions across sample groups via PERMDISP2 (the multivariate analogue of Levene's test, equivalent to vegan::betadisper) with 999 permutations (Table S6), and ran three sensitivity analyses on the same Kulczynski binary distance (Table S6): (i) pairwise balanced PERMANOVA in which each pair of sample groups was downsampled without replacement to the smaller group size, repeated 999 times; (ii) a zooplankton‐only model excluding water‐column samples, both at full sample size and with balanced downsampling to *n* = 68 per group across 999 iterations; and (iii) month‐adjusted PERMANOVA reported with marginal‐term significance (adonis2 with by = ‘margin’) and PERMANOVA with permutations restricted within sampling month (strata = month). Following Anderson and Walsh (2013), under unbalanced designs, PERMANOVA results are interpreted as testing whether some combination of centroid location and dispersion differs across groups.

#### 2.7.3 Community Structure Characterisation Relevant to Hypothesis 1

We quantified the Chao1—to estimate total genus richness, Pielou's evenness—to assess the equitability of fungal genus distributions, and Shannon–Wiener indices to provide an integrated measure of both richness and abundance for each zooplankton‐associated sample and the surrounding water. These metrics were calculated using mia::addAlpha on genus‐level counts transformed to relative abundance. Diversity indices were assessed using a non‐parametric Kruskal–Wallis test to determine if significant differences existed among the sample groups (Cladocera—body and gut, Copepoda, Rotifera, or water column). Subsequently, Dunn's post hoc tests were performed to identify specific pairwise differences between groups, with *p*‐values adjusted using the Sequential Bonferroni (Holm) correction to control for multiple comparisons. These statistical analyses were performed using the software Past 4.14 (Hammer et al. 2001). The resulting indices were summarised per host group and sampling month and visualised as time series with boxplots for the zooplankton groups and a line for the water‐column reference (Figure 3, Figures S1 and S5).

#### 2.7.4 Differential Prevalence and Abundance Testing (MaAsLin3)—Addresses Hypotheses 1, 2 and 3a

To identify which individual fungal genera, and which functional traits, are characteristic of specific zooplankton groups versus the water column (Hypothesis 1), shift with sampling month or are dominated by terrestrial taxa consistent with allochthonous inputs (Hypothesis 2), and to compare gut and body samples in *Daphnia* spp. (Hypothesis 3a: ingestion vs. persistent gut colonisation), we used MaAsLin3 (v. 0.99.6; Nickols et al. 2024). MaAsLin3 was chosen because it explicitly handles the zero‐inflation, compositionality and library‐size variability typical of amplicon datasets, and because it allows simultaneous adjustment for relevant covariates while reporting both prevalence (logistic) and abundance (linear) components. Models were fitted with total‐sum scaling (normalisation = ‘TSS’) and ln‐transformation (transform = ‘LOG’), with predictors standardised (standardise = TRUE). Library size (readsum_std), sample group and sampling month were included as fixed effects. Significance was assessed at max_significance = 0.05 using Benjamini–Hochberg correction within each factor and across all features (genera and genus‐associated traits; joint *q*‐value, qval_joint ≤ 0.05). For the prevalence sub‐model, results are reported as odds ratios: an OR > 1 indicates that a taxon is more likely to be present in the focal group than in the reference group, and the magnitude indicates how many times more likely. For example, an odds ratio of 3 relative to Cladocera means that the focal taxon is three times more likely to be found in the focal group than in the Cladocera reference group.

#### 2.7.5 Cross‐Validation of Differential Abundance (ANCOM‐BC2)

Because differential‐abundance methods can yield divergent results depending on their underlying assumptions (e.g., handling of zeros, normalisation, treatment of compositionality), the MaAsLin3 abundance results were additionally cross‐validated using ANCOM‐BC2 (Lin and Peddada 2024), with the same fixed effects, global Benjamini–Hochberg correction and the built‐in sensitivity analysis for pseudo‐count addition. Concordance between the two frameworks provides additional confidence that the detected taxon‐level signals are robust rather than method‐specific artefacts.

#### 2.7.6 Spore‐ and Cell‐Size Comparisons—Address Hypothesis 3b

Median mitospore and meiospore lengths per sample were used as predictors in the PERMANOVA models described above (Hypothesis 3b: size‐based prey selection). In addition, the statistical significance of differences in spore sizes among sample groups was assessed using the Wilcoxon signed‐rank test, chosen because spore‐size distributions are non‐normal and heterogeneous in variance, with Holm correction for multiple pairwise comparisons. Distributions of fungal spore and yeast cell volumes across host groups were summarised at the genus level using values curated from the literature and from the Aguilar‐Trigueros et al. (2023) dataset, log‐transformed for visualisation, and displayed alongside the differential prevalence results (Figure 5, Figure S6). Scripts used for data analysis are publicly available through https://figshare.com (DOI: 10.6084/m9.figshare.c.8266720).

## 3 Results

### 3.1 Diversity and Composition of Zooplankton‐Associated Fungi

**FIGURE 2:**
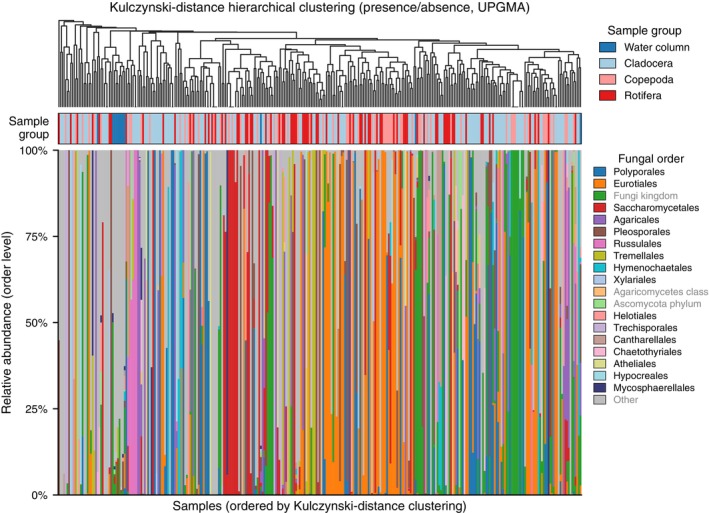
Hierarchical clustering and order‐level fungal community composition of zooplankton‐associated samples and ambient water‐column samples. Top panel: Dendrogram from Unweighted Pair Group Method with Arithmetic mean (UPGMA) hierarchical agglomerative clustering on Kulczynski binary distance based on order‐level presence/absence (all detected orders, *n* = 76). Middle: Sample‐group annotation strip in the same sample order as the dendrogram. Bottom: Stacked bars showing per‐sample relative abundance of the 19 most abundant fungal orders, with all remaining orders pooled as ‘Other’. Sample order along the x‐axis reflects the dendrogram leaves; samples within similar branches of the dendrogram have similar fungal‐order presence/absence profiles. Black labels indicate fungal orders; grey labels (e.g., ‘Fungi kingdom’, ‘Ascomycota phylum’, ‘Agaricomycetes class’, ‘Other’) refer to OTUs that could not be classified at the order level or showed lower relative abundance and were aggregated to ‘Other’.

After processing and quality filtering of raw reads and exclusion of samples with very low library size, a total of 21,836,094 fungal reads (mean 70,896 per sample) were obtained from 308 samples yielding 1701 OTUs covering 8 phyla, 28 classes, 60 orders, 128 families and 190 genera with 88%, 83%, 78%, 66%, 64% of OTUs classified at the respective level (Figure 2, Tables S3 and S4).

**FIGURE 3:**
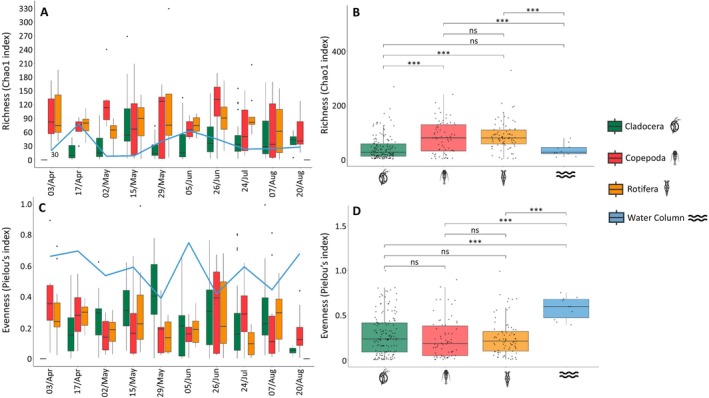
(A) Fungal genus richness at specific sampling points (Chao1 index) and associated with the three studied zooplankton groups and the water column (B). The evenness of samples (Pielou's index) at specific sampling points (C) and associated with the three studied zooplankton groups and the water column (D). Significance levels were determined using Dunn's post hoc tests with sequential Bonferroni (Holm) correction. The *p*‐values are represented by ***p < 0.001, **p < 0.01 and *p < 0.05.

Fungal genus richness (Chao1) associated with Cladocera did not differ from that observed in the surrounding water column. In contrast, fungal richness associated with Copepoda (*p‐*value = 0.019) and Rotifera (*p‐*value = 0.002) was significantly higher (Figure 3A,B, Table S5). No significant differences in evenness were detected among the zooplankton groups; however, all zooplankton groups differed significantly from the surrounding water (Figure 3C,D, Table S5—Cladocera *p‐*value = 2.82e^−5^; Copepoda *p‐*value = 1.81e^−5^; Rotifera *p‐*value = 5.02e^−6^). A similar trend was observed for overall α‐diversity (Figure S1, Table S5). Sample group was the strongest predictor of fungal assemblage composition in PERMANOVA based on genus‐level presence–absence Kulczynski dissimilarity (Table 1), though explained variation was modest (species level: *R* ^2^ = 6.5%; sample group: *R* ^2^ = 4.0%; both *p* = 0.001).

**TABLE 1:** PERMANOVA of fungal communities aggregated at genus level using binary transformation with Kulczynski distance.

Variable	Higher taxon			Species		
	*F*	*p*	*R* ^2^ (%)	*F*	*p*	*R* ^2^ (%)
Library size	0.93	0.535	0.33	1.01	0.421	0.36
Meiospores length	1.39	0.151	0.50	1.29	0.203	0.46
Mitospores length	3.60	**0.001**	1.28	3.49	**0.001**	1.24
Taxonomic level	3.79	**0.001**	4.05	1.82	**0.001**	6.47
Two‐days precipitation	1.66	**0.046**	0.59	1.68	**0.048**	0.60
Model	2.83	**0.001**	7.06	1.90	**0.001**	9.48
Residual	NA	NA	92.94	NA	NA	90.52

*Note:* Samples were either grouped with respect to the sample group or the species level of zooplankton. Of the tested variables, 2‐day mean precipitation, median spore length of mitospores, and zooplankton sample group/species were found to be highly significant (〈I〉p〈I〉 values in bold). Continuous variables were *z*‐score standardised. Library size was used as a control variable for sequencing depth‐related artefacts.

**FIGURE 4:**
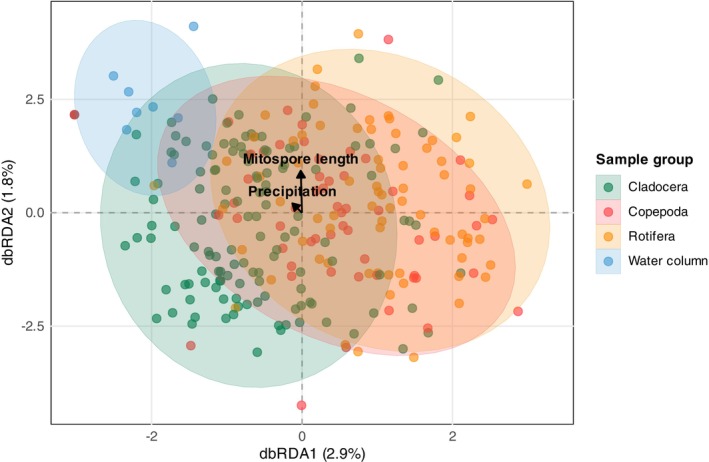
dbRDA of zooplankton‐ and water column‐associated fungi corresponding to the PERMANOVA in Table 1, with sample group as grouping variable, showing only the significant covariates of the PERMANOVA. Mitospore length = median mitospore length in each sample, Precipitation = two‐day mean precipitation (both *z*‐score standardised). X and Y axis labels indicate the explained variation (%).

**FIGURE 5:**
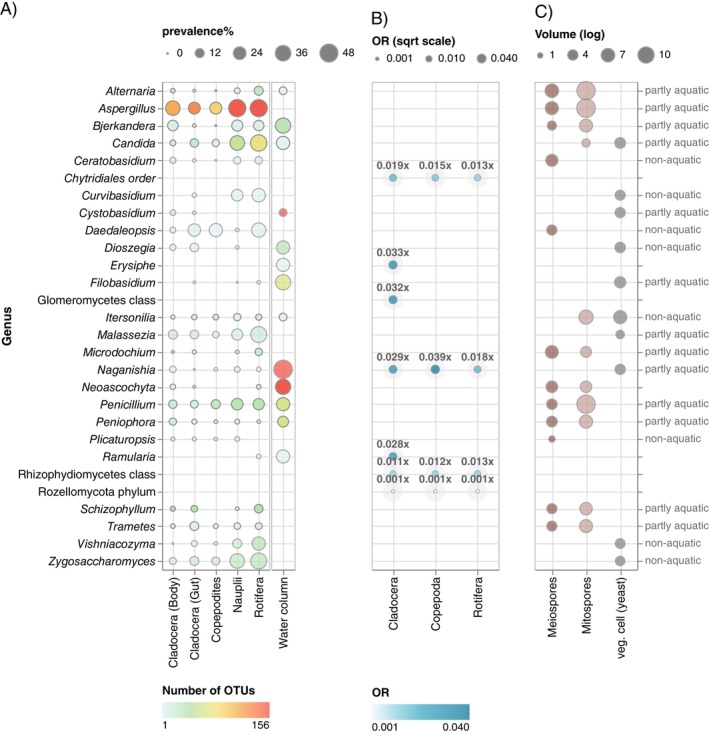
(A) Prevalence of the five most prevalent, abundant and OTU‐rich genera in each zooplankton group. (B) Differentially prevalent fungal taxa using the water column as reference. Light grey shadows provide an OR = 1 reference backdrop; coloured inner circles are scaled by OR using a square‐root scale and coloured by OR; grey labels show the exact OR. (C) log‐transformed mean spore volumes and mean cell volumes (yeasts). Y‐axis labels on the right side correspond to the potential of fungal genera to live in aquatic environments according to the FungalTraits database. OR, odds ratio.

Species identity explained more variation than sample group but had a lower *F*‐ratio, suggesting less distinct group separation at the species level; we therefore visualised community structure at the sample group level (Figure 4). In dbRDA ordination, Rotifera‐ and Copepoda‐associated communities overlapped substantially, whereas Cladocera‐associated communities clustered closer to the water column. Among the environmental covariates screened in PERMANOVA models that included sample group and rotating sets of ~5 covariates (see Materials and Methods for the full list), only 2‐day precipitation (*p* = 0.047, *R* ^2^ = 0.6%) and mitospore length (*p* = 0.001, *R* ^2^ = 1.3%) remained significant alongside sample group (Table 1). These effects were coherent across models, indicating statistically consistent yet biologically modest associations. Within‐group dispersions were heterogeneous (PERMDISP2 *F* = 14.79, *p* = 0.001; Table S6), with lower variance in pooled water‐column samples (mean within‐group Kulczynski distance 0.67) than in single‐specimen zooplankton samples (0.81–0.88), reflecting differences in sampling resolution rather than a methodological artefact. To probe whether this heterogeneity could fully account for the sample‐group effect, we conducted three sensitivity analyses (Table S6). Pairwise balanced PERMANOVA confirmed compositional differences in five of six contrasts (*p* < 0.05 in 995–999 of 999 iterations for Cladocera vs. Copepoda, Cladocera vs. Rotifera, Cladocera vs. water column, Copepoda vs. water column and Rotifera vs. water column); the Copepoda‐vs.‐Rotifera contrast was the sole weak signal (539 of 999 iterations significant; median *p* = 0.046), consistent with the substantial overlap of these two groups in the dbRDA ordination (Figure 4). Excluding water‐column samples to remove the pooled‐vs‐individual sampling contrast, the sample‐group effect among zooplankton groups remained significant in both the full (pseudo‐*F* = 4.34, *R* ^2^ = 2.85%, *p* = 0.001) and balanced‐resampled (999 of 999 iterations significant, median *p* = 0.001) tests. Month‐adjusted PERMANOVA (host group as a marginal term: pseudo‐*F* = 4.94, *R* ^2^ = 4.56%, *p* = 0.001) and PERMANOVA with permutations restricted within sampling month (pseudo‐*F* = 5.23, *R* ^2^ = 4.86%, *p* = 0.001) both retained the sample‐group signal, ruling out a purely seasonal‐sampling artefact. Following Anderson and Walsh (2013), we therefore interpret the PERMANOVA result as confirming that some combination of mean and dispersion differs across sample groups, with convergent evidence from the dbRDA (Figure 4) and the prevalence‐based MaAsLin3 analysis (Figure 5) supporting compositional distinctness rather than mere dispersion heterogeneity.

### 3.2 Functional Characterisation of Fungal Communities

Based on genus‐level information from the FungalTraits database, the majority of prominent fungal genera in the water column and zooplankton samples were semi‐aquatic yeasts (Figure 5, Figure S2A). The overall prevalence of yeasts in this dataset was substantially higher than in published soil datasets, whereas genera exhibiting filamentous mycelial growth were underrepresented in our study (Figure S2B). Fungal genera with a nectar/sap saprotrophic lifestyle (i.e., primarily feeding on sugar‐rich substrates such as nectar and tree sap, growing in yeast cellular form; Spencer et al. 1992; Lachance 2006) according to the FungalTraits database (Figure S2C) were substantially more prevalent in our dataset compared with published studies from terrestrial environments, further highlighting the significance of fungi with a yeast growth form.

Rotifers and the water column were most distinct in terms of functional fungal groups. Compared with Cladocera samples, the taxa classified as nectar/tap saprotrophic fungi, yeasts and animal‐associated fungi were significantly more prevalent in Rotifers (2.8, 2.9 and 2.6 times the odds, respectively, MaAsLin3 BH‐adjusted *q* ≤ 0.05). In contrast, fungi with closed hymenium were significantly more prevalent in the water column (7.5 times the odds, MaAsLin3 BH‐adjusted *q‐*value ≤ 0.05, Figure S2B). Closed hymenia have been noted as an evolutionary adaptation to aquatic environments in Dothideomycetes (Hyde et al. 2013), including the genera *Ramularia* and *Neoascochyta*, which were prominent in the water column in our dataset (Figure 5).

### 3.3 Differential Prevalence and Abundance Analysis

*Malassezia*, *Zygosaccharomyces, Daedaleopsis* and *Aspergillus* were the most prevalent and characteristic genera in zooplankton samples and were not detected in the water column. For the water column, the most characteristic fungal taxa were members of the Rozellomycota, which we were not able to classify at a lower taxonomic level (absent from zooplankton samples, but present in over 90% of water column samples, > 100 times the odds), members of the Rhizophydiomycetes (> 20 times the odds relative to zooplankton occurrence), Chytridiales (> 15 times the odds compared to zooplankton) and the genus *Naganishia* (> 10 times the odds relative to zooplankton). Comparing the water column and Cladocera‐associated fungal assemblages, the fungal taxa *Erysiphe*, *Ramularia* and Glomeromycetes spp. were significantly more prevalent in the water column (all > 10 times the odds relative to Cladocera; Figure 5). Copepoda and Rotifera shared a set of fungal taxa (mostly yeasts), which were significantly less prevalent in Cladocera: *Candida* (3.4 times and 4.1 times the odds relative to Cladocera), *Curvibasidium* (7.5 times and 10.6 times the odds), Eurotiomycetes (4.7 times and 4.2 times the odds) and *Zygosaccharomyces* (3.7 times and 4.4 times the odds). *Vishniacozyma* was predominantly found in Rotifers (1.9 times the odds compared to Cladocera, Figure S3). No significant differences in the abundance or prevalence of fungal taxa were found among zooplankton species, *Daphnia* spp. body and gut samples, or between Copepoda adults and Copepoda juveniles. Overall seasonality did not significantly shape fungal community composition. However, two highly prevalent individual genera did show seasonal patterns: *Daedaleopsis* and *Vishniacozyma* were progressively more abundant from April and peaked in July, with 4096‐fold and 256‐fold changes, respectively (MaAsLin3 BH‐adjusted *q‐*values of 2.4e^−2^ and 8.4e^−2^, Figure S4).

### 3.4 *Daphnia* spp. Gut and Body‐Associated Fungi

**FIGURE 6:**
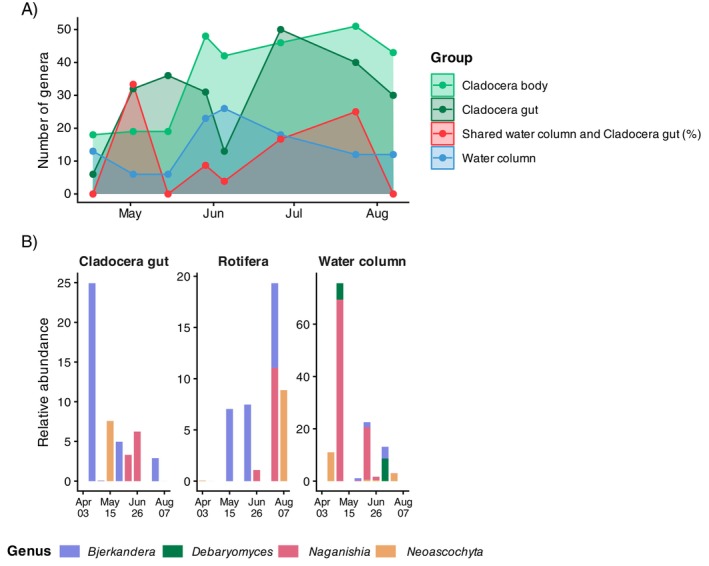
(A) Number of genera in Cladocera body and gut, water column samples on each sampling date, and the number of shared genera between water column and Cladocera gut. (B) Stacked plot of relative abundance (%) of the main shared genera between water column and Cladocera gut.

We found no significant differences in overall fungal genus richness, diversity, or evenness among *Daphnia* spp. gut and body samples (Figure S5, Table S7). The proportion of water column‐associated fungal genera found in *Daphnia* guts varied over time: no shared taxa were observed in April (the start of sampling) and the beginning of August, while 28.6% of water‐column‐associated genera were found in *Daphnia* guts in early May; from early to late summer, we observed a gradual increase peaking in late July (Figure 6). The majority of the genera identified in both the guts and the water column were common environmental yeasts (*Candida*, *Debaryomyces*, *Dioszegia*, *Filobasidium*, *Itersonilia*, *Naganishia* and *Sporobolomyces*); the rest belonged to polypore (*Bjerkandera*) or micro‐filamentous fungi (*Neoascochyta*, *Penicillium*, *Phaeosphaeria* and *Stemphylium*). *Daphnia* spp. gut and body samples did not significantly differ in terms of associated fungal species‐derived prevalence‐weighted spore sizes (matched on sampling date; Table S8).

### 3.5 Spore‐ and Cell‐Size Effects

The length, width and volume of fungal mitospores and meiospores in the water column were significantly larger than those observed in zooplankton samples (Figure S6). We included the median spore lengths of mitospores and meiospores matched at the fungal species level from the Aguilar‐Trigueros et al. (2023) dataset as fixed effects in the PERMANOVA model (Table 1). This extended our set of significant variables and added 1.28% and 1.24% of explained variation in fungal assemblages associated with sample groups and at the zooplankton species level, respectively.

## 4 Discussion

Our results indicate that the fungal assemblages associated with zooplankton are distinct from those in the water column, characterised by high genus richness, particularly in Copepoda and Rotifera, but lower diversity and evenness. This pattern contrasts with bacteria, whose assemblages tend to have significantly higher richness in the water column than those associated with zooplankton hosts (see, for example, De Corte et al. 2018; Wall et al. 2025). Fungi tend to be present at significantly lower abundances in the water column than bacteria (Sen et al. 2023). Therefore, the high genus richness observed in zooplankton samples may reflect the range of fungi and their propagules that the zooplankton feed on, while the dominance of a few taxa suggests selective feeding on abundant or preferred propagules. Zooplankton feeding may concentrate fungal propagules from diverse sources, including terrestrial inputs, generating elevated genus richness relative to the surrounding water.

As in published studies on bacterial microbiomes (Eckert et al. 2021), variability in fungal assemblages among individual animals of the same species was high. Regardless of the variation at the level of individual animals, the zooplankton taxonomic group and species identity were significant factors structuring the zooplankton‐associated fungi. The low reported *R* ^2^ values in our PERMANOVA are typical for single‐specimen microbiome datasets that retain inter‐individual heterogeneity (Datta et al. 2018), in contrast to pooled designs that average out stochastic noise and tend to yield larger apparent effect sizes (Engel et al. 2012; Kelly et al. 2015). We did not find any significant differences in fungi associated with the two developmental stages of copepods in our study, despite indications from the literature of distinct prey preferences (Brucet et al. 2008). The multivariate dispersion diagnostic indicated that within‐group dispersion was heterogeneous (PERMDISP2 *F* = 14.79, *p* = 0.001; Table S6). Mean within‐group Kulczynski distances were lower in the pooled water column samples (0.67) than in the single‐specimen zooplankton samples (0.81–0.88), which is consistent with the different sampling resolution. Following Anderson and Walsh (2013), we therefore interpret the sample‐group PERMANOVA as evidence that a combination of centroid location and dispersion differs among groups; the dbRDA and prevalence‐based differential testing provide convergent support that the groups are compositionally distinct rather than simply differentially variable.

Based on the widely accepted ‘mycoloop’ theory (Kagami et al. 2014), we expected to find zoosporic fungi associated with zooplankton, especially in the guts of Cladocera. However, while this group of fungi was highly prevalent in the surrounding water column, it was absent from the zooplankton samples, indicating that feeding on zoospores did not occur during the studied period.

On the other hand, a majority of the genera identified in both Cladoceran guts and the water column were environmental yeasts typical for environments rich in organic nutrients, as well as polypore or micro‐filamentous genera known for their high spore production in terrestrial environments and for long‐distance spore dispersal (Junninen and Komonen 2011; Golan 2017). Most identified fungal genera were classified as either partly aquatic—that is, capable of growth at the water–terrestrial interface according to the FungalTraits database—or as non‐aquatic (terrestrial) taxa, primarily soil or wood saprotrophs and plant pathogens.

It is technically challenging to distinguish among epibiotic colonisers, gut microflora, potential pathogens or endosymbionts, and microbes assimilated as food particles, particularly in studies using next‐generation sequencing, which has become the primary approach in microbiome analysis. Many studies attempt to distinguish between epibiotic and gut‐colonising microorganisms using surface‐sterilisation techniques. However, it is not known how effective these are, and there is evidence that the gut microbiome is affected by this treatment (Binetruy et al. 2019). Large zooplankton species are suitable for gut dissection, and this approach has been used in marine settings on mesozooplankton taxa (Hannes and Sommaruga 2008). Here, only the largest cladoceran species from the genus *Daphnia* were found suitable for gut dissection. As we could not reliably ensure that only epibionts are affected by surface sterilisation (cf. Yu et al. 2022), we report data on whole‐body (surface + gut) microbiomes for the other smaller zooplankton taxa analysed here.

We did not observe significant differences in fungal assemblages between the dissected *Daphnia* body and gut samples. This may be because the body samples likely contained fungal spores or cells lodged in the feeding combs, which could be considered food particles and thus were also found in the guts. The absence of taxa uniquely associated with the gut makes it unlikely that yeast cells colonise the *Daphnia* digestive tract.

Zooplankton are frequently infected by fungal pathogens. When selecting zooplankton for analysis under the dissection microscope, we sought individuals without visible signs of disease or parasitism. In our dataset, we have not found any of the better‐described pathogenic fungi, such as *Metschnikowia bicuspidata* (Sun et al. 2023), or members of the genera *Catenaria*, *Coelomomyces*, or *Olpidium* (Gleason et al. 2010).

Taken together, the lack of fungal hyphae or colonies on the bodily surfaces under microscopic observations, the limited surface area for fungal colonisation (especially in rotifers), the absence of gut‐specific taxa, and the significant effects of fungal spore size suggest that most fungi detected in zooplankton samples represent potential prey. The observed among‐taxon and among‐individual variation, therefore, likely reflects zooplankton prey size constraints and preferences, as well as the pronounced spatiotemporal dynamics of the system (Vrba et al. 2024). In further support of the predator–prey association between zooplankton and fungi, our data show that the Cladocera‐associated fungal assemblages are more similar to the water column assemblages than are those associated with Copepoda and Rotifera. This is in line with published research showing Cladocera to be more passive feeders with low prey selectivity, and Copepoda and Rotifera exhibiting a relatively high degree of discrimination between high and low quality of food particles, as well as the ability to ingest sessile prey from larger particles suspended in the water column (DeMott 1986; Kiørboe 2011; Gilbert 2022).

Of all the environmental and biotic factors analysed, only rainfall had a significant effect on fungal assemblage structure. Most of the variables analysed belong to the standard set commonly considered in limnological studies and are known to primarily structure free‐living planktonic microbes, particularly bacteria, algae and bacterivorous protozoa. In contrast, planktonic fungi—especially those occurring epibiotically on hosts or transiently in the water column following dispersal from the watershed—are likely structured by different environmental and host‐related factors (De Corte et al. 2018) that are not routinely measured in plankton ecology. These may include processes in the watershed, particulate organic matter characteristics and degradability, host surface properties, moulting frequency and immune responses. Consequently, zooplankton may act as strong ecological filters, decoupling their associated fungal assemblages from ambient limnological conditions in the water column (Wall et al. 2025). The significance of rainfall and mitospore length, together with the high prevalence of soil‐ and plant‐associated fungal taxa in our dataset, suggests that zooplankton—particularly Cladocera—may at least partly exploit allochthonous fungi and their propagules entering the water column from the terrestrial environment (Magyar et al. 2021). While fungal cells in yeast form are readily assimilated by zooplankton under culture conditions (Manklinniam et al. 2018), it remains to be experimentally confirmed whether fungal propagules such as meiospores and mitospores are efficiently digested, whether fungi constitute a meaningful nutritional resource for freshwater zooplankton, and under which environmental conditions.

## 5 Conclusions

Our results show that the fungal assemblages associated with freshwater zooplankton reflect host taxonomy and are temporally dynamic. The dominance of known terrestrial saprotrophs, the absence of zoospore‐associated fungi in zooplankton samples, and the lack of gut‐specific taxa all point towards a trophic association supplemented by allochthonous fungal input via atmospheric deposition or runoff. These findings suggest an unexplored route for cross‐ecosystem carbon and nutrient flow (cf. LeBrun et al. 2018) and highlight non‐zoosporic fungi as a potential food source for zooplankton. This may be especially relevant within the context of global change‐related predictions indicating that increased terrestrial runoff into freshwater, coastal and estuarine environments will significantly impact planktonic food web dynamics (Adamczuk et al. 2019; Osakpolor et al. 2021).

## Author Contributions

D.S., V.K., C.P., J.V. and MŠ designed the research. J.V., V.K., D.S. and C.P. collected samples. J.S., C.P. and D.S. analysed the data and drafted the manuscript. V.K. performed gut excisions, zooplankton identification and enumeration. M.Š. and J.V. provided supporting environmental and biotic data. All authors contributed to the final version of the manuscript.

## Funding

This work was supported by Grantová Agentura České Republiky (project no. 23‐06429S).

## Disclosure

Benefits Generated: This research was co‐created by researchers from three Czech institutions: Biology Centre CAS, University of South Bohemia, and Mendel University Brno. The benefits from this collaboration result from the public sharing of these data and findings.

## Conflicts of Interest

The authors declare no conflicts of interest.

## Supporting information


**Figure S1:** The diversity of fungal assemblages (Shannon index) of zooplankton and water column samples over time (A) and associated with the three studied zooplankton groups (B). Significance levels were determined using Dunn's post hoc tests with sequential Bonferroni (Holm) correction. The *p*‐values are represented by ***p < 0.001, **p < 0.01 and *p < 0.05.
**Figure S2:** Comparison of 133 studies and > 5500 soil samples from the GlobalFungi database with our dataset (zooplankton) in respect to the proportion (%) of fungal genera and their associated aquatic potential (A, top), growth form (B, middle) and primary lifestyle (C, bottom). NA indicates unknown classification.
**Figure S3:** Differentially prevalent (A) fungal taxa within the studied zooplankton groups and (B) functional traits according to the FungalTraits database in all groups including water column using Cladocera as reference. Only groups with differentially prevalent taxa/traits are shown. All *q*‐values ≤ 0.05.
**Figure S4:** Seasonality of two zooplankton associated fungal genera: *Daedaleopsis* and *Vishniacozyma* relative abundances increase towards and peak in July. *q*‐value 2.4e^−2^ and 8.4e^−2^, respectively, with an lfc (log fold change) of 8.0 and 12.0 in July and April, respectively.
**Figure S5:** Comparison of ecological indices among *Daphnia* spp. body, extracted guts and water column samples. Significance levels were determined using Dunn's post hoc tests with sequential Bonferroni (Holm) correction. The *p*‐values are represented by ***p < 0.001, **p < 0.01 and *p < 0.05.
**Figure S6:** Length (A, top), width (B, middle) and volume (C, bottom) of mitospores and meiospores in the sample groups. Statistical significance was assessed using Wilcoxon test (variables are non‐normally distributed and heterogeneous) with Holm correction, *p*‐value thresholds are indicated by asterisks: *p* ≤ 0.05 (*), *p* ≤ 0.01 (**) and *p* ≤ 0.001 (***).

**Table S1:** Sample information (zooplankton species, broad range of zooplankton group, sampling time, month time and location), and physical, chemical, and biotic conditions characterising the pelagic environment at each sampling point.
**Table S2:** Cell shape and average cell length (μm), width (μm) and volume (μm^3^) for each fungal genus group, with corresponding references from the scientific literature.
**Table S3:** Fungal operational taxonomic units (OTUs) distribution across all studied samples.
**Table S4:** Taxonomic classification for each fungal OTU obtained from all studied samples.
**Table S5:** Statistical comparison of fungal genus diversity indices (Chao1 richness, Pielou's evenness and Shannon–Wiener diversity) among zooplankton groups (Cladocera, Copepoda and Rotifera) and the water column. The values in the first column represent the chi‐square (χ^2^) statistic followed by the *p*‐value derived from Kruskal–Wallis tests in order to compare the groups. Pairwise comparison values represent *p*‐values obtained from Dunn's post hoc tests adjusted using the Sequential Bonferroni (Holm) correction.
**Table S6:** Beta‐diversity dispersion and PERMANOVA sensivity analyses. Input data for all analyses were genus‐level presence/absence tables with Kulczynski distance. Unless otherwise noted, tests used 999 permutations.
**Table S7:** Statistical comparison of fungal genus diversity indices (Chao1 richness, Pielou's evenness and Shannon–Wiener diversity) between *Daphnia* spp. body and gut samples relative to the water column. The values in the first column represent the chi‐square (χ^2^) statistic followed by the *p*‐value derived from Kruskal–Wallis tests. Pairwise comparison values represent *p*‐values obtained from Dunn's post hoc tests adjusted using the Sequential Bonferroni (Holm) correction.
**Table S8:** Prevalence‐weighted sample and sampling date‐matched spore size statistics.

## Data Availability

Raw amplicon sequence data can be accessed through the European Nucleotide Archive (PRJEB94073); scripts used for data analysis can be downloaded from https://figshare.com (DOI: 10.6084/m9.figshare.c.8266720).
