# Supplementary material for: Fungi Associated With Freshwater Zooplankton Are Taxon‐Specific, Temporally Dynamic and Reflect Allochthonous Inputs
Source: Mol Ecol. 2026 Jul 24;35(14):e70436. doi: 10.1111/mec.70436 (PMC13398034; doi:10.1111/mec.70436)
Supplement: Supplementary file 1 — Figure S1: The diversity of fungal assemblages (Shannon index) of zooplankton and water column samples over time (A) and associated with the three studied zooplankton groups (B). Significance levels were determined using Dunn's post hoc tests with sequential Bonferroni (Holm) correction. The p‐values are represented by ***p < 0.001, **p < 0.01 and *p < 0.05. Figure S2: Comparison of 133 studies and > 5500 soil samples from the GlobalFungi database with our dataset (zooplankton) in respect to the proportion (%) of fungal genera and their associated aquatic potential (A, top), growth form (B, middle) and primary lifestyle (C, bottom). NA indicates unknown classification. Figure S3: Differentially prevalent (A) fungal taxa within the studied zooplankton groups and (B) functional traits according to the FungalTraits database in all groups including water column using Cladocera as reference. Only groups with differentially prevalent taxa/traits are shown. All q‐values ≤ 0.05. Figure S4: Seasonality of two zooplankton associated fungal genera: Daedaleopsis and Vishniacozyma relative abundances increase towards and peak in July. q‐value 2.4e−2 and 8.4e−2, respectively, with an lfc (log fold change) of 8.0 and 12.0 in July and April, respectively. Figure S5: Comparison of ecological indices among Daphnia spp. body, extracted guts and water column samples. Significance levels were determined using Dunn's post hoc tests with sequential Bonferroni (Holm) correction. The p‐values are represented by ***p < 0.001, **p < 0.01 and *p < 0.05. Figure S6: Length (A, top), width (B, middle) and volume (C, bottom) of mitospores and meiospores in the sample groups. Statistical significance was assessed using Wilcoxon test (variables are non‐normally distributed and heterogeneous) with Holm correction, p‐value thresholds are indicated by asterisks: p ≤ 0.05 (*), p ≤ 0.01 (**) and p ≤ 0.001 (***). [file MEC-35-e70436-s001.docx]

**Fungi associated with freshwater zooplankton are taxon-specific, temporally dynamic, and reflect allochthonous inputs**

Johannes Schweichhart^1,2^, Caio César Pires de Paula^1,2^, Veronika Kreidlová^2^, Jaroslav Vrba^1,2^, Michal Šorf^3^, and Dagmara Sirová^1*^

^1^Institute of Hydrobiology, Biology Centre CAS, České Budějovice, Czech Republic

^2^Faculty of Science, University of South Bohemia, České Budějovice, Czech Republic

^3^Faculty of AgriSciences, Mendel University in Brno, Brno, Czech Republic


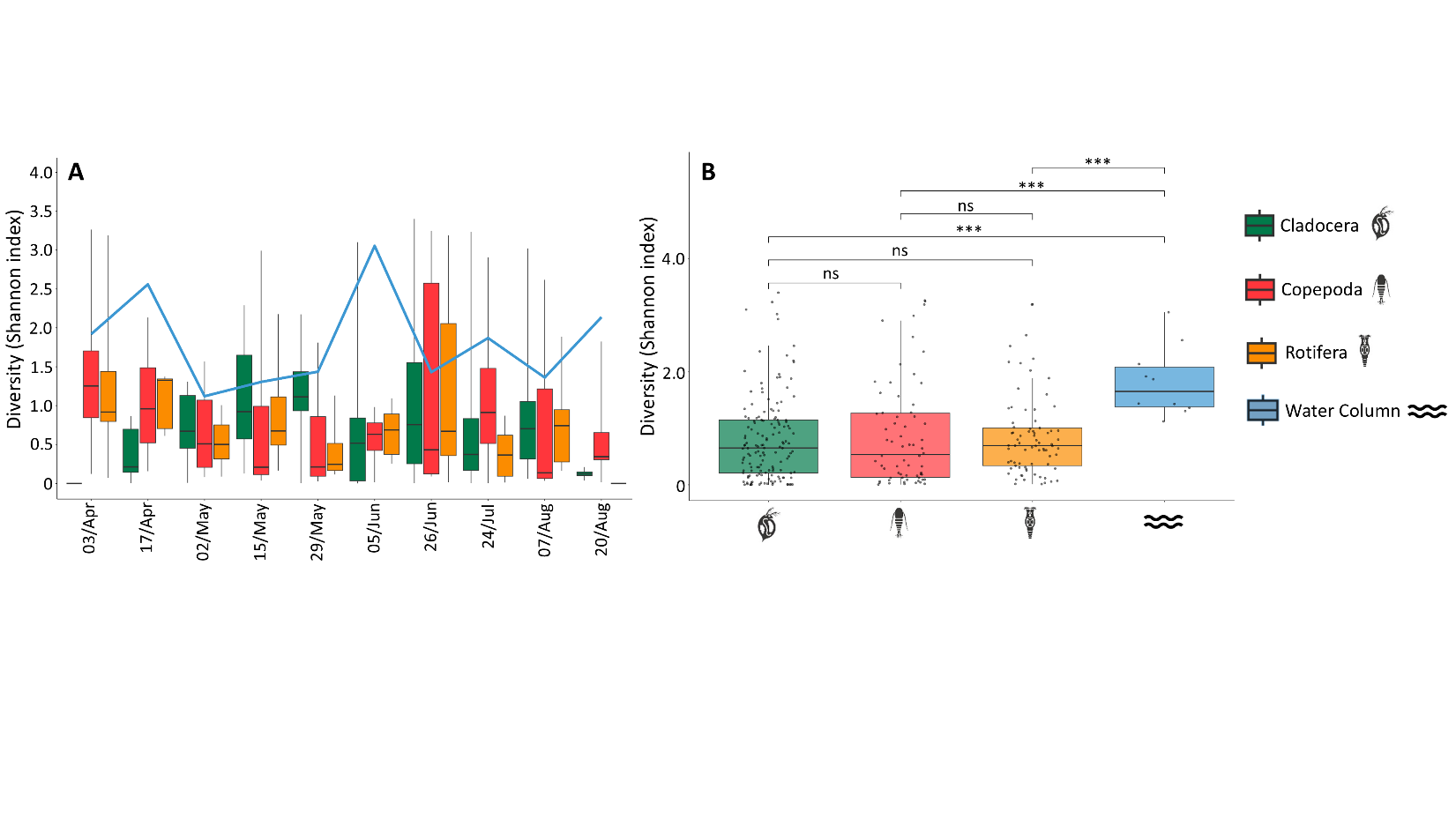
**Figure S1**. The diversity of fungal assemblages (Shannon index) of zooplankton and water column samples over time (**A**) and associated with the three studied zooplankton groups (**B**). Significance levels were determined using Dunn’s post hoc tests with sequential Bonferroni (Holm) correction. The p-values are represented by “***” = p < 0.001,”**” = p < 0.01, and “*” = p < 0.05.


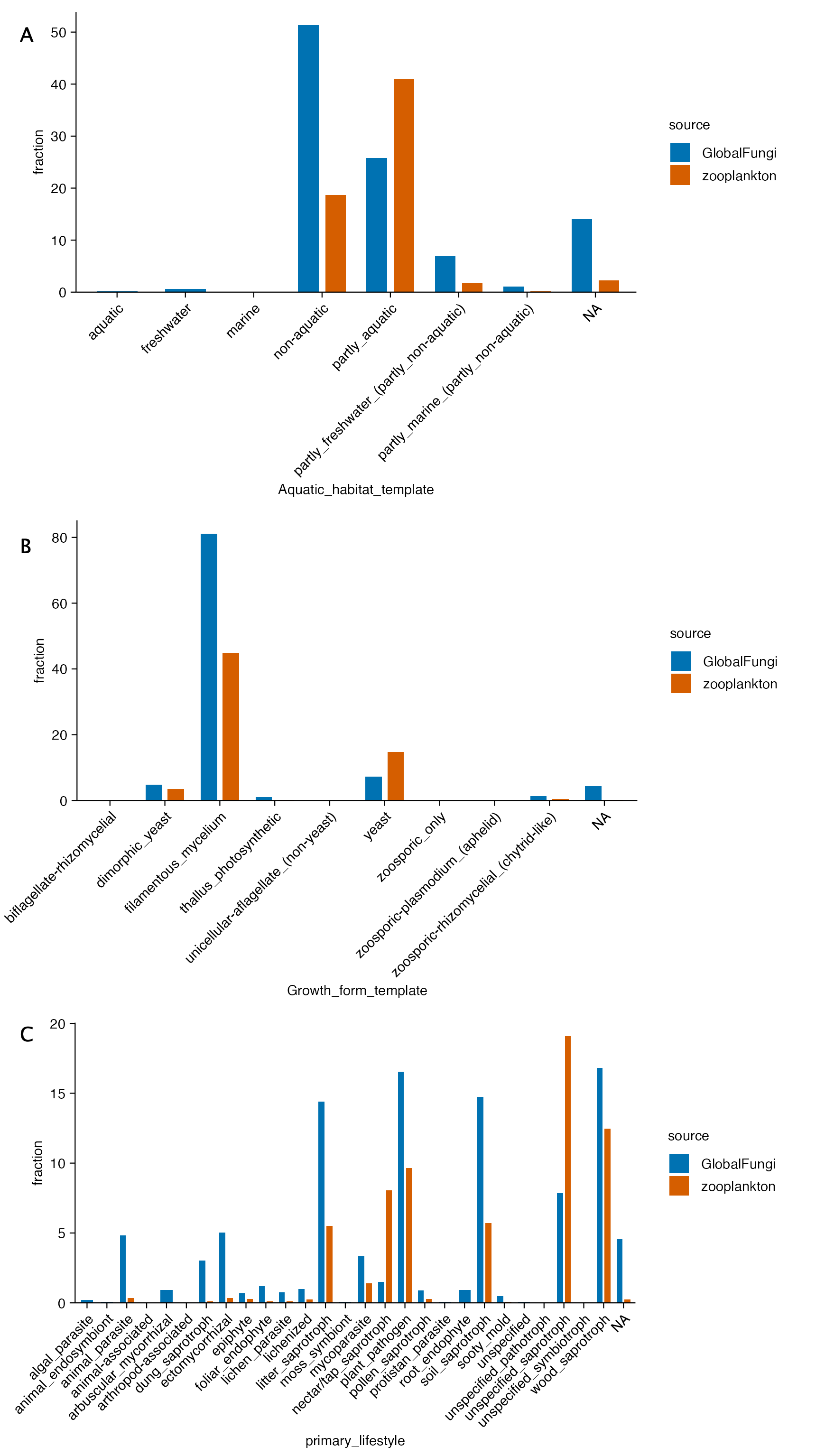


**Figure S2** comparison of 133 studies and >5500 soil samples from the GlobalFungi database with our dataset (zooplankton) in respect to the proportion (%) of fungal genera and their associated aquatic potential (**A, top**), growth form (**B, middle**), and primary lifestyle (**C, bottom**). NA indicates unknown classification.


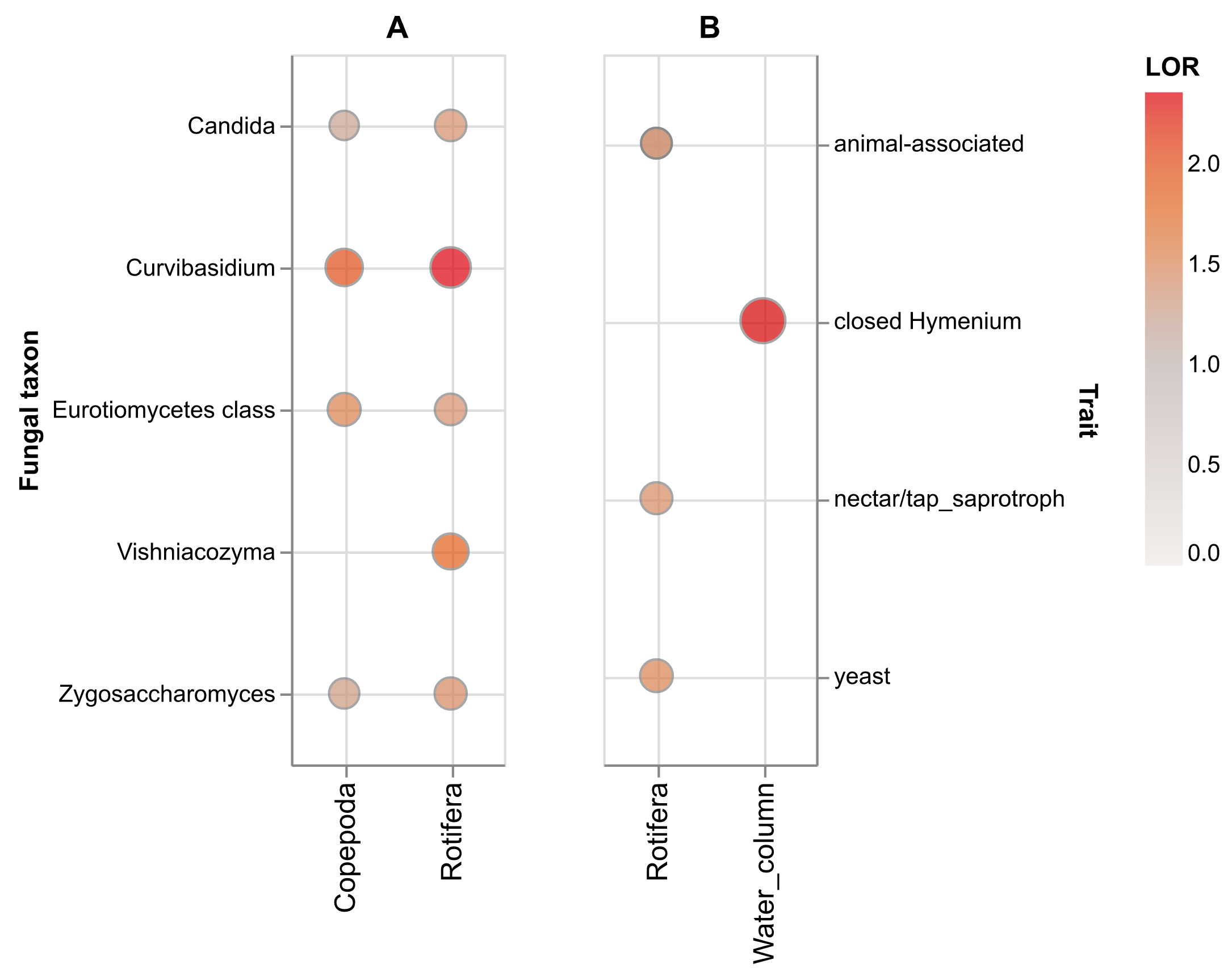


**Figure S3** Differentially prevalent (**A**) fungal taxa within the studied zooplankton groups and (**B**) functional traits according to the FungalTraits database in all groups, including the water column, using Cladocera as a reference. Only groups with differentially prevalent taxa/traits are shown. All *q*-values <= 0.05.


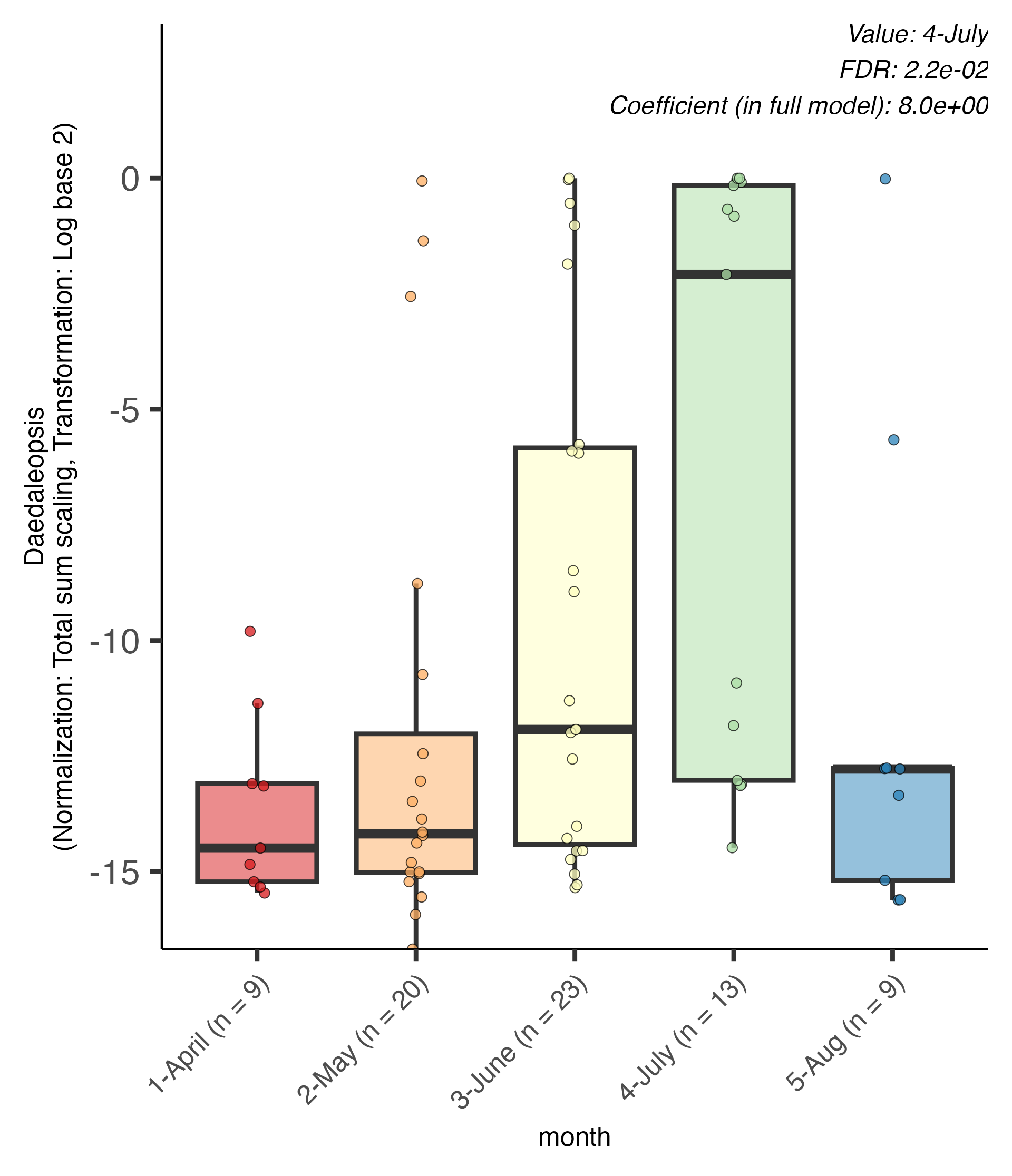


**Figure S4** Seasonality of two zooplankton-associated fungal genera: *Daedaleopsis* and *Vishniacozyma* relative abundances increase towards and peak in July. *q*-value 2.4e^-2^ and 8.4e^-2^, respectively, with an lfc (log fold change) of 8.0 and 12.0 in July and April, respectively.

**
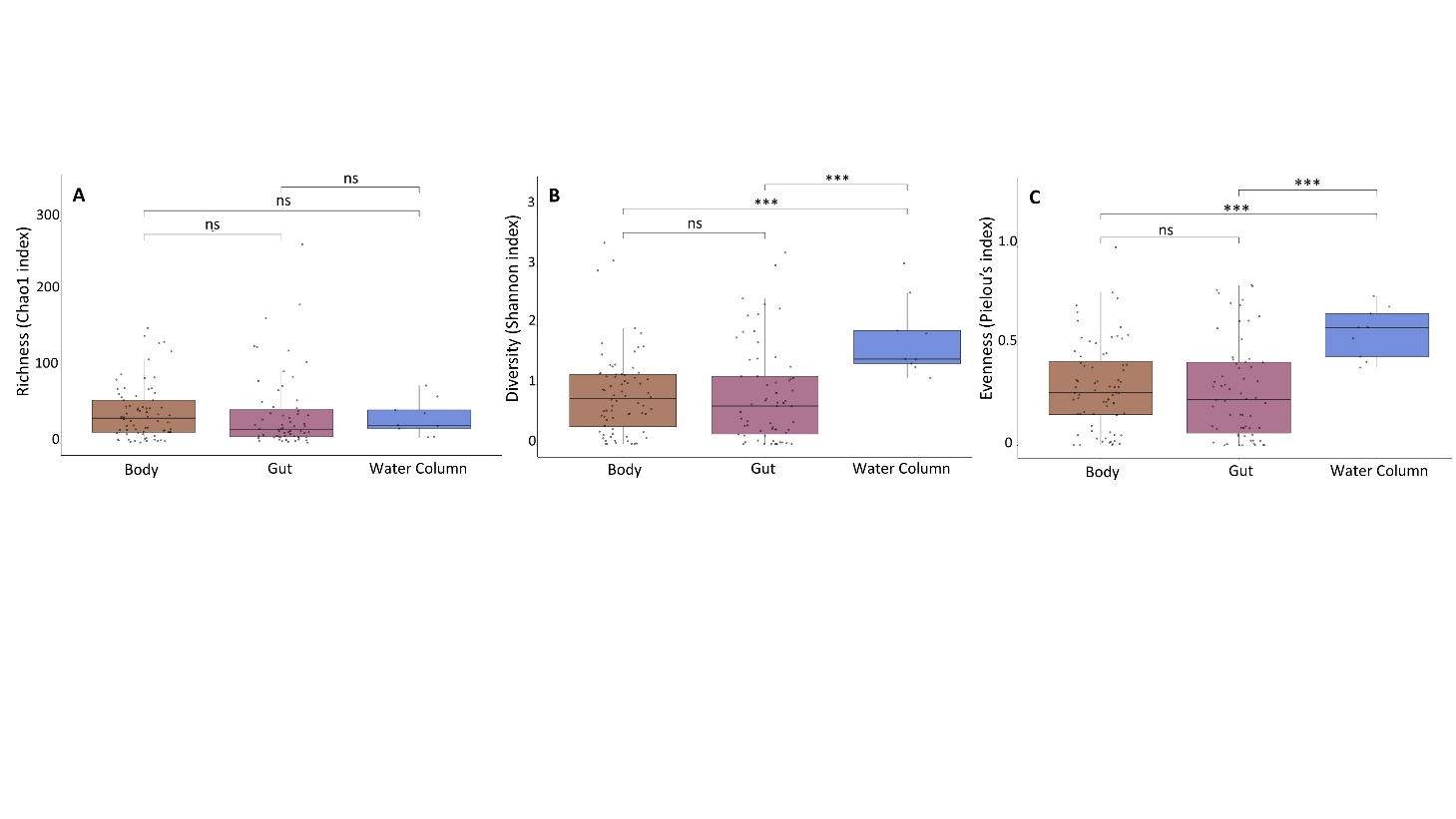
**

**Figure S5** Comparison of ecological indices among *Daphnia* spp. body, extracted guts, and water column samples. Significance levels were determined using Dunn’s post hoc tests with sequential Bonferroni (Holm) correction. The p-values are represented by “***” = p < 0.001,”**” = p < 0.01, and “*” = p < 0.05.


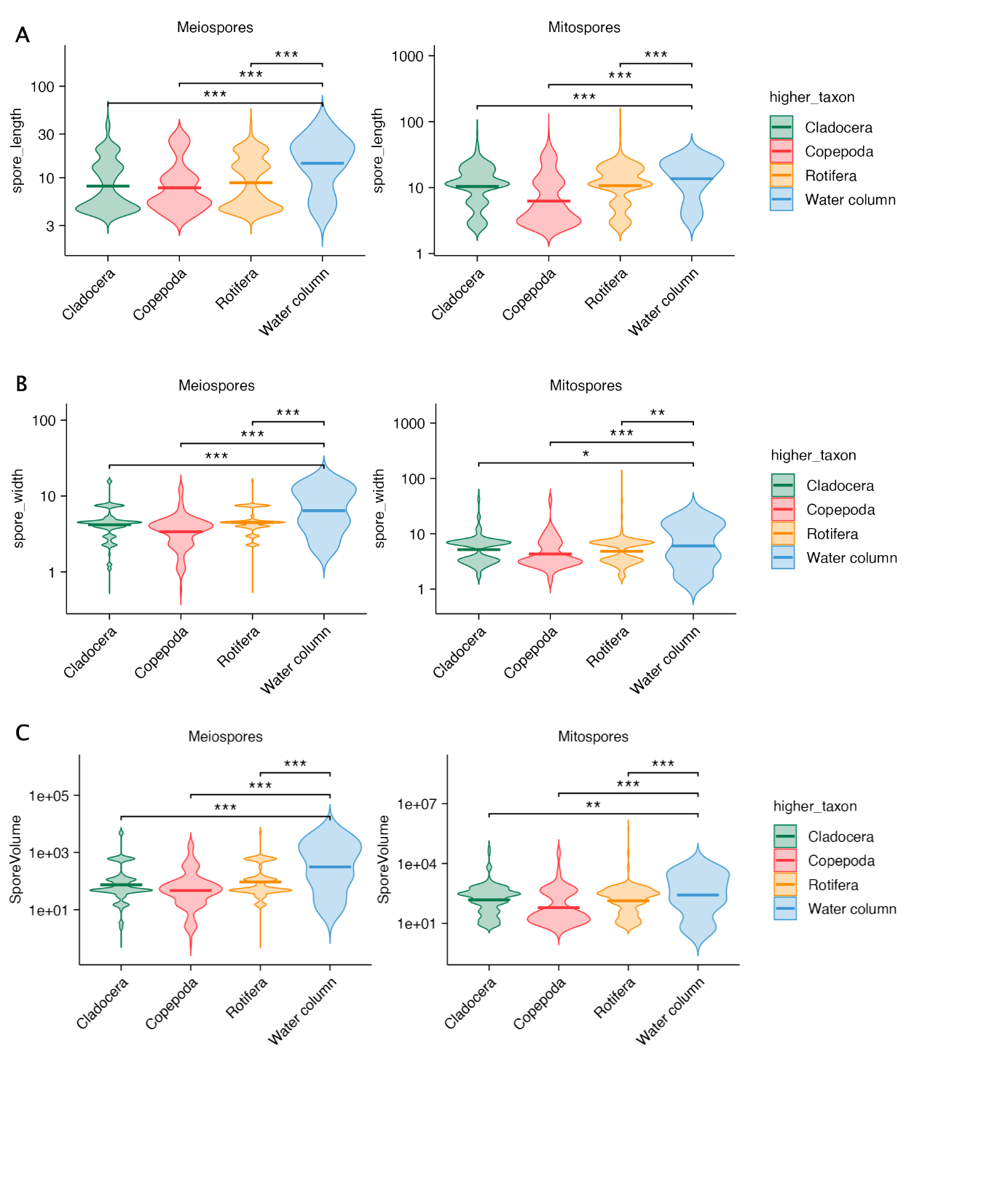


**Figure S6** Length **(A, top)**, width **(B, middle),** and volume **(C, bottom)** of mitospores and meiospores in the sample groups. Statistical significance was assessed using Wilcoxon test (variables are non-normally distributed and heterogeneous) with Holm correction, *p*-value thresholds are indicated by asterisks: *p* ≤ 0.05 (*), *p* ≤ 0.01 (**), and *p* ≤ 0.001 (***).
